# Evidence of slightly increased Pol II pausing in UPF1-depleted *Drosophila melanogaster* cells

**DOI:** 10.17912/micropub.biology.000319

**Published:** 2020-10-16

**Authors:** Anand K Singh, Jie Zhang, Daniel Hebenstreit, Saverio Brogna

**Affiliations:** 1 University of Birmingham, UK; 2 University of Warwick, UK

**Figure 1:**
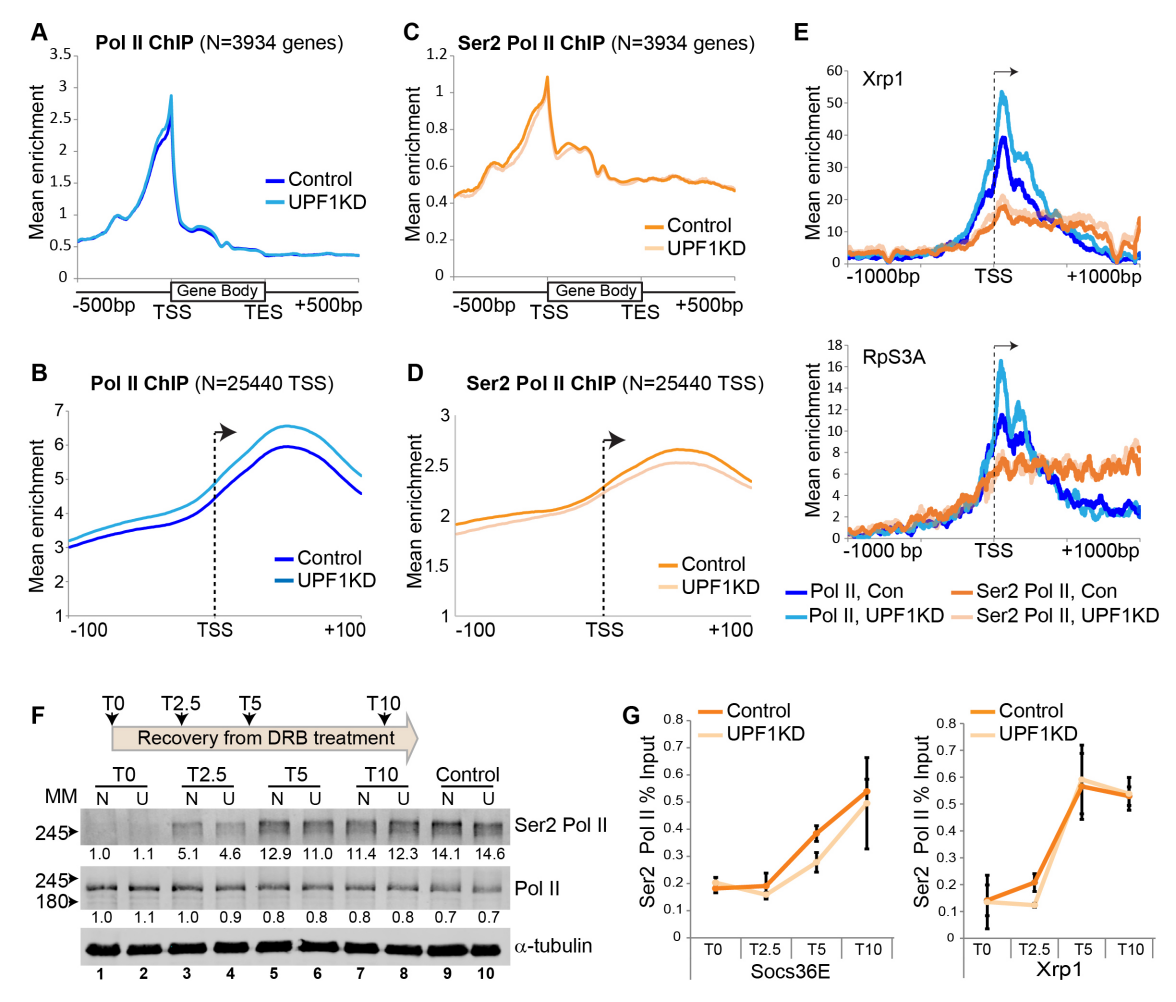
**Depletion of UPF1 increases Pol II pausing at TSS proximal sites**. (**A**) Metagene line plots of ChIP-seq data showing average occupancy of unphosphorylated Pol II at gene bodies (scaled as 16 bins of full length) and + 500 bp from either end, in normal S2 cells (control, dark blue) or UPF1-RNAi S2 cells (UPF1KD, light blue); based on 3934 (N) transcription units that do not overlap within 500 bp of either end. (**B**) Metagene plots showing unphosphorylated Pol II at TSS +/- 100 bp, based on 25440 TSSs that are not closer than 200 bp. (**C**) Metagene plots as in **A** showing Ser2 Pol II occupancy; in normal S2 cells (control, dark orange line) and UPF1-RNAi S2 cells (UPF1KD, light orange line). (**D**) As for **B** but showing the Ser2 Pol II profiles. (**E**) Normalised ChIP-seq profile of either Pol II (blue lines) or Ser2 Pol II (orange lines) at the *Xrp1* gene, +/- 1000 bp from the TSS (transcript, NM_001275790). The equivalent plots for *RpS3A* (NM_166714) gene are shown below. Dark lines refer to normal S2 cells, light lines to UPF1-RNAi, as indicated in the legend below the plots. (**F**) Schematics of the timeframe (top) of transcription recovery following removal of DRB after 1 hour (T0) treatment of S2 cells; protein levels were assayed at T0 and at three time points from DRB removal: 2.5 minutes (T2.5), 5 minutes (T5) and 10 minutes (T10). The panel below shows western blotting of Ser2 Pol II (top row) and unphosphorylated Pol II (middle row) at different time points after DRB washout in normal (N) and UPF1-RNAi (U) cells. The α-tubulin was detected as a loading control (bottom row). Values below lanes are ImageJ quantifications of the Ser2 Pol II and unphosphorylated Pol II band/s intensities, which are normalised by the signal of the α-tubulin band in the same lane and expressed as ratios of the intensity of the corresponding band in lane 1. (**G**) Line graphs show real-time PCR quantifications of average Ser2 Pol II enrichment at either *Socs36E* gene (on left) or *Xrp1* gene (on right) following DRB treatment and removal at different time points as described in **F**. The PCR primers used are located about 4 kb downstream of the TSS of both *Socs36E* and *Xrp1* genes.

## Description

UPF1 is an RNA helicase that scans RNA to unwind secondary structures and to displace associated factors (Franks **et al.*,* 2010; Fiorini **et al.*,* 2015; Lee **et al.*,* 2015; Kanaan *et al.*, 2018). UPF1 has been mostly studied for its role in nonsense mediated mRNA decay (NMD) and other translation-dependent RNA quality-control pathways in the cytoplasm (Isken and Maquat, 2008; Kim and Maquat, 2019). However, we have recently reported that UPF1 associates, genome wide, with nascent transcripts at most Pol II transcription sites in *Drosophila* (Singh *et al.*, 2019). The association of UPF1 with nascent transcripts appears to be necessary for the release of processed polyadenylated mRNAs from their transcription sites and also for their export from nucleus to cytoplasm (Singh **et al.*,* 2019).

Here we considered whether the association of UPF1 with nascent transcripts influences Pol II transcription, and tested it by ChIP-seq of Pol II in *Drosophila* S2 cells. We discovered (using two biological replicates) that there is significantly more unphosphorylated Pol II associated with the start of genes in cells depleted of UPF1 (**Figure 1A and 1B**). This difference is most apparent 20 to 60 base pair (bp) downstream of the transcription start site (TSS), as shown in the expanded depiction in **Figure 1B** which is based on many more individual TSSs. This is measured by quantifying the aggregate Pol II signal over a +/- 100 bp span at each of 25440 TSSs (P = 0.011). A 1.2-fold or greater increase of unphosphorylated Pol II in UPF1-depleted cells was detected at 1990 TSSs. The 20-60 bp downstream region corresponds to TSS-proximal Pol II pausing sites in *Drosophila* (Adelman and Lis, 2012). Conversely, the amount of Ser2 Pol II associated with these TSSs was unchanged or marginally reduced in UPF1-depleted cells (**Figure 1C and 1D**). The increase in unphosphorylated Pol II loading downstream of the TSS in UPF1-depleted cells, alongside fairly constant Ser2 Pol II loading, is illustrated here by *Xrp1* and *RpS3A* (**Figure 1E**), two genes that are highly transcribed and show strong UPF1 association in normal cells (Singh **et al.*,* 2019).

We considered whether this increase in unphosphorylated Pol II at pausing sites might be a consequence of reduced Ser2 phosphorylation, resulting in the slow release of Pol II from pausing sites and abnormal transcription elongation. We tested this hypothesis by examining the reappearance of newly phosphorylated Ser2 Pol II following withdrawal of 5, 6-Dichlorobenzimidazole 1-β-D-ribofuranoside (DRB), which is a transcription inhibitor that blocks Ser2 phosphorylation and should lead to rapid depletion of Ser2 Pol II. As expected, DRB treatment of S2 cells causes a drastic depletion of Ser2 Pol II and an increase of unphosphorylated Pol II (**Figure 1F**, compare control lanes 9 and 10 with DRB-treated lanes 1 and 2). Ser2 Pol II levels began to recover soon after DRB removal and were similar to those of untreated control cells within 10 minutes (**Figure 1F**, compare lanes 7-8 with control lanes 9-10). However, the recovery in UPF1-depleted cells seemed slower during the first 2.5 minutes and to a lesser extent after 5 minutes (**Figure 1F**, compare lanes 3 *vs*. 4 and 5 *vs*. 6). Similar observations were made in another independent replicate of the experiment. A comparable blunted recovery of gene-associated Ser2 Pol II in UPF1-depleted cells was detected by ChIP-PCR at the two representative highly active genes, *Socs36E* and *Xrp1* (**Figure 1G**), both of which show strong association with UPF1 (Singh **et al.*,* 2019). RpS3A was not examined because it is too short (about 2 kb) for this assay.

These observations indicate that UPF1 might, by associating with nascent transcripts, influence Ser2 phosphorylation of Pol II at TSS-proximal pausing sites and hence transcription elongation at several *Drosophila* genes. Alternatively, or additionally, UPF1 might affect premature transcription termination and Pol II release from DNA downstream of the TSS, which are other, perhaps more important determinants of the high accumulation of Pol II at the beginning of genes (Kamieniarz-Gdula and Proudfoot, 2019) .

## Methods

**Cell culture and RNA interference**

S2 cells (CVCL_Z232, laboratory stock originally purchased from Invitrogen/Thermo Fisher Scientific) were cultured in Insect–XPRESS media (Lonza) supplemented with 10% Fetal Bovine Serum (FBS) and 1% Penicillin-Streptomycin-Glutamine mix (P/S/G, Invitrogen) at 27°C. To make the RNAi constructs for UPF1, the specific sequences were PCR amplified from S2 cell genomic DNA by using corresponding primer pairs. Along with the desired gene sequence, the primer pair carried the T7 promoter sequence at their 5’ end. The amplified PCR fragments were purified using Monarch® PCR and DNA Cleanup Kit (T1030S, NEB) and dsRNA was synthesized using the T7 RiboMAX express RNAi system (P1700, Promega). To induce RNAi, a six-well culture dish was seeded with 10^6^cells/well in serum-free media and mixed with 15 µg of dsRNA/well. Following 1 hour incubation at room temperature, 2 mL of complete media was added to each well and the cells were incubated for the next three days to knockdown UPF1 before harvesting.

**Western blotting and antibodies**

Western blotting was performed as described previously (Singh **et al.*,* 2019). The antibodies used in western blotting were diluted as follow: Rabbit anti-Ser2 Pol II (Abcam, ab5095, 1:5000), Mouse anti-Pol II (Abcam, ab817 (8WG16), 1:5000) and mouse anti-β-tubulin (Sigma-Aldrich, T5168 1:5000).

**DRB treatment**

S2 cells were treated with DRB (Sigma-Aldrich, 125µM) for 1 hour at room temperature. Ser2-Pol II ChIP was performed as described below and quantified by real-time PCR using *Socs36E* and *Xrp1* primers shown below.

**ChIP-seq and data analysis**

Chromatin immunoprecipitation, sequencing and data analysis was done as described previously (Singh **et al.*,* 2019). For each ChIP, typically we used 5 µg of antibody including Rabbit anti-Ser2 Pol II (Abcam, ab5095) and Mouse anti-Pol II (8WG16, Abcam, ab817). ChIP-signal quantification and metagene analysis were performed as previously described (Singh **et al.*,* 2019). All new ChIP-seq raw sequencing FASTQ data and Bedgraph files were deposited in the GEO repository (Accession No GSE157612), all other datasets used in this study were previously deposited (Accession No GSE16808).

**Primers List:**

UPF1RNAi-FP – 5’-**TTAATACGACTCACTATAGGGGAGA**GGAGAAGCCAGGCATTGA-3’

UPF1RNAi-RP – 5’-**TTAATACGACTCACTATAGGGGAGA**GACCGTGGCCCAACAGG-3’

(Bold is T7 promoter sequence)

Socs36E-FP – 5’-CAGAAAACCGCACACAGACA -3’

Socs36E-RP – 5’-CACACATCGGACTAACAGCG-3’

Xrp1-FP – 5’-TCATAATGCTTGTGGGGCCT -3’

Xrp1-RP – 5’-AGGGTCCCTCTAAACAAGCT-3’

## Reagents

See Methods
